# Examining the Impact of Puberty on Primary Headache Disorders in Female Schoolchildren: A Cross-Sectional Study

**DOI:** 10.7759/cureus.49871

**Published:** 2023-12-03

**Authors:** Nora Almuslim, Aeshah Alnajjar, Nurah Alkhteeb, Mashael Alhussain, Hanan Alrubaia, Ahmed Alkhateeb

**Affiliations:** 1 Neurology, King Faisal University, Al-Hofuf, SAU; 2 Medicine, King Faisal University, Al-Hofuf, SAU; 3 General Practice, King Faisal University, Al-Hofuf, SAU

**Keywords:** eastern region, school children, female, puberty, headaches disorder

## Abstract

Introduction and aim

Headaches are one of the most prevalent childhood disorders. Primary and secondary headaches are the two types of headaches affecting kids and teenagers. The three most typical primary headache forms are tension-type headaches (TTH), migraine, and cluster headaches. This study sought to determine the relationship between puberty and types of headaches.

Methods

This cross-sectional study was conducted from May 18 to July 31, among female schoolchildren aged between eight and 15 years in the Eastern Province of Saudi Arabia. Respondents were recruited through face-to-face interviews. A self-administered questionnaire was utilized, mainly consisting of demographic data and questions related to diagnosing and managing the impact of puberty in girls on the prevalence of primary headache disorder.

Results

In total, 481 female schoolchildren were interviewed, mostly between 13 and 15 years old (65.9%). Last year prevalence of headaches was 65.5%, with a significant difference among those who lived in Dhahran (p=0.001) and those with a family history of headaches (p<0.001). The most common type of headache was frequent TTH (16.4%) and chronic TTH (16%). Chronic TTH (p<0.001), frequent TTH (p<0.001), and migraine without aura (p=0.005) were significantly more common among the older age groups.

Conclusion

There was a high prevalence of headaches among female schoolchildren, with frequent TTH and chronic TTH being the most common. Furthermore, increasing age was associated with an increasing risk for chronic TTH, frequent TTH, and migraine headaches without aura. More epidemiological studies are necessary to determine the underlying causes of headaches among schoolchildren.

## Introduction

Headaches are defined as head pain that occurs anywhere in the head or neck areas [1]. Pain is a feeling that necessitates and requires medical attention. In accordance with the International Association for the Study of Pain, pain is "a distressing sensory and emotional experience related to actual or potential tissue damage, or described in terms of such damage" [2]. They can be a substantial source of concern for children and families, making them one of the top concerns at pediatrician and urgent care visits [3].

The prevalence of headaches rises throughout childhood, peaking in both sexes between the ages of 11 and 13 years [4]. By the time they turn 15 years old, 75% of kids are said to have major headaches, making headaches one of the most prevalent childhood disorders [5]. Headaches are more common in boys younger than seven years of age than in girls, but after puberty the ratio shifts in favor of girls [6]. Primary and secondary headaches are two categories of headaches. The International Classification of Headache Disorders clearly outlines the classification of headaches. Primary headaches involve those that are in a diseased state by themselves, whereas secondary headaches are those that are immediately brought on by or made worse by a known or assumed etiology [7].

Primary and secondary headaches are the two types of headaches that affect kids and teenagers [5]. Based on a differentiation between primary and secondary headaches, this categorization was developed. The tension-type headache, migraine, and cluster headache are the three most typical forms of primary headache [8]. Among the most common headache disorders, tension-type headache (TTH) and migraine were the main causes of headaches [9]. In both general practice and specialized neurology outpatient clinics, headache is a prevalent reason for medical consultations [10].

Despite being one of the top 10 causes of disability, headache is the most frequent neurological symptom, currently, it has a poor healthcare and public profile, which is linked to under-treatment and under-recognition [11]. The third leading contributor to disability is migraine, which can be so bad as to affect everyday activities and quality of life. The trigeminovascular theory is the most widely recognized pathophysiological explanation for migraine [12]. In adults with migraine, menstrual-related migraine is well known, but in young girls, this monthly pattern may start to appear during puberty and prepuberty, preceding the onset of the first menstrual cycle [7]. The pathogenesis of TTH appears to involve muscular components, notably those originating from the pericranial muscles, stress, and central sensitization; even so, given that the source of TSH is still unknown [13]. Although the pathophysiology of cluster headache has not been fully analyzed, potential mechanisms have been described. These include genetic predisposition, involvement of the trigeminovascular and cranial parasympathetic nervous systems, as well as the role of the central nervous system, especially the hypothalamic region, which is thought to play a crucial role in the genesis of the attacks [14]. High rates of absence from school due to headache that is also linked to a number of coexisting diseases, particularly in the neurological, mental, and cardiovascular systems, especially anxiety and depression, epilepsy, sleep issues, and ADHD [15]. This study aimed to examine the relationship between puberty and types of headaches.

## Materials and methods

This cross-sectional study was conducted from May 18, 2023, to July 31, 2023, and focuses on the relationship between primary headache disorder prevalence among female schoolchildren in the Eastern Province Region of Saudi Arabia and the onset of puberty in girls during the year 2022-2023. The distribution of the survey was facilitated with the aid of 10 data collectors from different universities in the Eastern Province Region to ensure the presence of enough participants to represent the population. For determining the prevalence of a health issue and analyzing its link with particular variables at a particular time, cross-sectional studies are the best option.

The study was conducted within the Eastern Province, which includes a number of cities and districts, in the cities of Al-Ahsa, Dammam, and Al-Khobar City. Due to its geographic diversity and the high prevalence of primary headache problems among female schoolgirls described in other studies, this region was chosen. The study was conducted in four secondary schools in the Eastern province of Al-Ahsa, Dammam, and Al-Khobar City were hosting the study. These schools were chosen based on their desire to take part and their ability to accommodate an adequate number of female students. Well-trained physicians conducted face-to-face interviews with the participants which were held on the school grounds in a safe and quiet location, guaranteeing comfort and secrecy.

All secondary schoolgirls included in this study, who are aged between eight and 15 years in accordance with the Ministry of Health guidelines, were randomly selected.

Inclusion and exclusion criteria

Female students between the ages of eight and 15 years, students who have consented after being fully informed, and students who are willing to take part in in-person interviews were included in this study. Students who fall outside of the mentioned age range, students who have a history of serious illnesses that might affect the outcomes of the study, and students who are unable or unwilling to give consent after being informed were excluded from the study.

Estimated response rate, confidence time period, required precision, and expected prevalence of primary headache disorders in female schoolgirls were all taken into consideration when calculating the sample size. The sample size was determined in order to provide enough statistical power. Richard Geiger was used to determine the sample size. The required sample size was calculated as 385 and the representative sample size was calculated using the following statistical formula:

 $n= P(1-P)\times (Za/2)2\times (E)2$

However, we covered a large population size to ensure proper representation of the sample. A total of 481 children were included in the study. The rights and well-being of the participants in medical research are protected under ethical considerations. The following ethical problems (which examined how puberty affects the prevalence of primary headache problems among female schoolchildren in the Eastern Region) were taken into consideration while this study was conducted: informed consent, confidentiality and privacy, free will, and minimal harm. The SPSS version 26 (Armonk, NY: IBM Corp.) was used to analyze the statistical data for this project. Categorical values were shown as numbers and percentages. The relationship between the incidence of headaches and the socio-demographic characteristics of female schoolchildren has been conducted using the chi-square test. We used the chi-square test to determine the association between the age group and the type of headache as well. A p<0.05 is determined as the statistical significance level.

## Results

A total of 481 female schoolchildren were enrolled. Table 1 describes the participants' demographic characteristics. Nearly two-thirds (65.9%) were aged between 13 and 15 years. The vast majority (62%) of the respondents lived in Al-Hasa city. Approximately 61.1% were at the secondary school level. A family history of headaches was reported by 35.1%.

**Table 1:** Basic demographic characteristics of the female schoolchildren (n=481)

Study variables	N (%)
Age group	
8-12 years	164 (34.1%)
13-15 years	317 (65.9%)
Residence city	
Al-Hasa	298 (62.0%)
Dammam	65 (13.5%)
Dhahran	23 (04.8%)
Jubail	43 (08.9%)
Qatif	19 (04.0%)
Al-Khobar	33 (06.9%)
Level of education	
Primary school	187 (38.9%)
Secondary school	294 (61.1%)
Family history of headache	
Yes	169 (35.1%)
No	312 (64.9%)

In Table 2, last year's prevalence of headaches was 65.5%. Among those who had headaches (n=315), 43.5% had a headache duration of four to seven days for at least two to four hours (38.1%) per day. Further, 26.7% had at least a monthly episode of headaches during the past year and an average headache severity (56.8%). The most common site of headache was the frontal (38.7%), and 52.4% were bilateral. Also, 45.4% experienced pressure/tightening headaches, with approximately 5-7 intensity.

**Table 2:** Prevalence and characteristics of headache among female schoolchildren (n=481)

Variables	N (%)
Have you had a headache over the past year?	
Yes	315 (65.5%)
No	166 (34.5%)
How many days did you have a headache? (n=315)	
1-3 days	130 (41.3%)
4-7 days	137 (43.5%)
>7 days	48 (15.2%)
Is there an impact on the academic level because of the headache? (n=315)	
Yes	181 (57.5%)
No	134 (42.5%)
Duration of headache in hours (n=315)	
Not mentioned	52 (16.5%)
≤1 hour	112 (35.6%)
2-4 hours	120 (38.1%)
≥5 hours	31 (09.8%)
How many bouts of headaches during the past year? (n=315)	
Not mentioned	30 (09.5%)
Once	20 (06.3%)
2-4 times	70 (22.2%)
5-10 times	72 (22.9%)
Monthly	84 (26.7%)
Weekly	39 (12.4%)
Severity of headache (n=315)	
Not mentioned	26 (08.3%)
Able to live with it and it does not affect daily activities	61 (19.4%)
Average sharpness	179 (56.8%)
Too severe to carry on with daily activities	49 (15.6%)
Headache site (n=315)	
Not mentioned	27 (08.6%)
Frontal	122 (38.7%)
Temporal	59 (18.7%)
General	43 (13.7%)
Orbital	32 (10.2%)
Vertical	20 (06.3%)
Occipital	12 (03.8%)
Bilateral or unilateral (n=315)	
Not mentioned	28 (08.9%)
Bilateral	165 (52.4%)
Unilateral	122 (38.7%)
Characteristics of headache (n=315)	
Not mentioned	27 (08.6%)
Pressure/tightening	143 (45.4%)
Throbbing/pulsating	119 (37.8%)
Stabbing	26 (08.3%)
Intensity of headache (n=315)	
<5	58 (18.4%)
5-7	202 (64.1%)
8-10	55 (17.5%)

**Figure 1:**
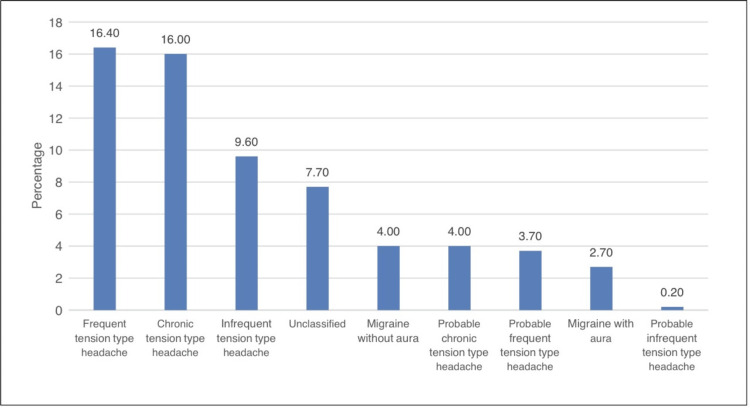
Bar chart illustrating the distribution of different headache types

In Figure 1, the most common type of headache was frequent TTH (16.4%), followed by chronic TTH (16%) and infrequent TTH (9.6%).

In Table 3, the incidence of headaches was significantly more common among those who lived outside Al-Hasa (p=0.001) and those with a family history of headaches (p<0.001).

**Table 3:** Relationship between the incidence of headache and the basic demographic characteristics of female schoolchildren (n=481) *P-value has been calculated using chi-square test. **P<0.05 has been considered significant.

Factor	Incidence of headache		p-Value*
	Yes, n (%) (n=315)	No, n (%) (n=166)	
Age group			
8-12 years	100 (31.7%)	64 (38.6%)	0.134
13-15 years	215 (68.3%)	102 (61.4%)	
Residence city			
Al-Hasa	181 (61.4%)	117 (76.5%)	0.041**
Dammam	47 (72.3%)	18 (27.7%)	
Dhahran	19 (82.6%)	04 (17.4%)	
Jubail	33 (76.7%)	10 (23.3%)	
Qatif	15 (78.9%)	04 (17.4%)	
Al-Khobar	20 (60.6%)	13 (39.4%)	
Level of education			
Primary school	116 (62.0%)	71 (38.0%)	0.203
Secondary school	199 (67.7%)	95 (32.3%)	
Family history of headache			
Yes	133 (78.7%)	36 (21.3%)	<0.001**
No	182 (58.3%)	130 (41.7%)	

In Table 4, the older age group was more associated with having chronic TTH (p<0.001), frequent TTH (p<0.001), and migraine without aura (p=0.005).

**Table 4:** Relationship between age group and the type of headache *P-value has been calculated using chi-square test. **P<0.05 has been considered significant.

Type of headache	Age group		p-Value*
	8-12 years, n (%) (n=164)	13-15 years, n (%) (n=317)	
Chronic tension-type headache	06 (03.7%)	71 (22.4%)	<0.001**
Frequent tension-type headache	09 (05.5%)	70 (22.1%)	<0.001**
Infrequent tension-type headache	10 (06.1%)	36 (11.4%)	0.063
Migraine with aura	04 (02.4%)	09 (02.8%)	0.798
Migraine without aura	01 (0.60%)	18 (05.7%)	0.005**
Probable chronic tension-type headache	05 (03.0%)	14 (04.4%)	0.623
Probable frequent tension-type headache	04 (02.4%)	14 (04.4%)	0.279
Probable infrequent tension-type headache	01 (0.60%)	0	0.164
Unclassified	15 (09.1%)	22 (06.9%)	0.389

## Discussion

This study explored the prevalence of primary headache disorder and how it affects female schoolchildren at the age of puberty. The findings of this study revealed that the one-year prevalence of primary headaches was 65.5%. This is almost consistent among the schoolchildren in Kuwait, with primary headache prevalence of 47.98%, while the one-year prevalence of migraine was 23.6%, with the lifetime prevalence said to be 84.9% [16]. Consistent with our report, the prevalence of recurrent headaches among Turkish children aged between 12 and 17 years was 52.2% [17]. The lowest prevalence of headaches found among schoolchildren has been reported by Laurell et al. [18], with a prevalence of 9.8% and 11% of TTH and migraine headache types detected among schoolchildren aged between seven and 15 years. 

Data in our study suggests that female schoolchildren living in Dhahran with a family history of headaches were at significant risk factors for primary headaches. Significant risk factors for headaches vary widely according to the sample population. For example, Shuabi et al. found that girls were more associated with primary headaches compared to boys among middle schoolers, but no differences were found between genders in relation to primary school students [1]. According to the reports of Al Jumah et al., the higher frequency of primary headaches in middle school females was attributed to female sex hormones and puberty [9]. Migraine was inversely correlated with age >45 years, while probable medication-overuse headache (pMOH) was prevalent among those aged 46-55 years. Also, increasing levels of education were associated with increasing risk for TTH. Additionally, some studies relate migraine headaches to psychological disorders, such as the study done [15,19,20].

The most common type of headache was frequent TTH (16.4%), followed by chronic TTH (16%), and the third was infrequent TTH (9.6%). Other headache types were migraine without aura (4%), probable frequent TTH (4%), migraine with aura (2.7%), and the least of them were probable infrequent TTH (0.2%). In Austria, 37% had episodic migraine without aura, 22% had episodic migraine with aura, and episodic and chronic TTH were 12% and 8%, respectively, and the least was chronic migraine (2%) [10]. Among medical students in the Western Region, frequent TTH was the most common (31%), followed by probable infrequent TTH (24%), and probable frequent TTH was the third (11.3%) [12]. Researchers highlighted the high prevalence of headaches among medical students, which they assumed was higher than the general population affecting academic performance that requires public health attention.

Stratifying by age (younger: aged 8-12 years vs. older: aged 13-15 years), we noted that the older age groups were at significant risk for having chronic TTH, frequent TTH, and migraine without aura. Other types of headaches were almost identical in results between the younger and the older age groups. According to the reports of Shuaibi et al., female middle schoolers were more associated with the diagnosis of migraine headache and TTH [1], which was consistent with the study of Al Jumah et al. reporting migraine and pMOH were associated with female respondents [9].

Moreover, we ought to underline the characteristics of our sample population diagnosed with primary headaches. For instance, 43.5% of this group of population had four to seven days of every headache episode for approximately two to four hours per day. For the last year, 26.7% said that headaches occurred monthly, mostly frontal headaches (38.7%) and bilateral (52.4%), with pressure and tightening being the most common (45.4%). Though 56.8% indicated average sharpness severity and with 5-7 headaches intensity, a great proportion of the headache population (57.5%) expressed that the level of headaches had affected their academic performance. These findings highlight the importance of special attention needed to be given to this group of population. In Taif, nausea occurred in more than one-third of patients before and during headache encounters, with 47% of them having pain scores ranging from 4-6 [12]. Among schoolchildren in Bangladesh, 53.3% had moderate severity of headaches, while 19.9% were severe [19]. The children also reported experiencing nausea and vomiting (47.2%) and photophobia (24.7%) during headaches.

Limitations

Our study has various shortcomings that might be addressed in follow-up research. First, the interview was conducted in a school, far from a headache clinic where the majority of the patients had mild headaches, interfering with the outcome. Second, because the target age range is between eight and 15 years, we had the same communication challenges with this age group. Some of them may have misunderstood the questions and provided misleading responses.

## Conclusions

Almost two-thirds of female schoolchildren suffered headaches, with frequent TTH and chronic TTH being the most common. Respondents with a family history of headaches had a higher risk of suffering from any form of headaches. However, increasing puberty age was associated with increasing risk for chronic TTH, frequent TTH, and migraine without aura. Headache is a major health issue among schoolchildren. With growing age, girls more frequently suffer from migraines and other forms of chronic headaches. This study necessitates further epidemiological research.
